# Identification of a tumor microenvironment-related gene signature for predicting prognosis in patients with gastric cancer

**DOI:** 10.1097/MD.0000000000044032

**Published:** 2025-08-29

**Authors:** Xuelian Li, Yi Xie, Fanghui Zhou, Pailan Peng

**Affiliations:** a Department of Gastroenterology, The Affiliated Hospital of Guizhou Medical University, Guizhou, China; b Guizhou Medical University, Guizhou, China.

**Keywords:** gastric cancer, immunoinfiltrating cell, nomogram, prognosis, TME

## Abstract

Tumor microenvironment (TME) plays an important role in the prognosis of gastric cancer (GC). The aim of this study was to identify a TME-related gene signature and provide a basis for prognosis evaluation of GC. X-cell and cluster analyses were performed on 373 tumor samples from The Cancer Genome Atlas-Stomach Adenocarcinoma. Prognostic-related genes were screened using differential analysis. Univariate Cox analysis, LASSO regression, and multivariate Cox analysis were used to determine the candidate genes and construct the prognostic model. Independent prognostic and correlation analyses of clinical characteristics were performed. In the observational study, patients were divided into high- and low-risk groups according to the expression levels and risk coefficients of the 4 model genes. Mutation characteristics, immune cell differences, and TME differences between the high- and low-risk groups were analyzed. The expression levels of these key genes were subsequently validated using reverse transcription quantitative polymerase chain reaction and Western blotting. Two hundred and twenty-five candidate genes were obtained by differential analysis. Performed in the training set, and 4 hub genes (CTHRC1, APOD, S100A12, and ASCL2) were finally determined as prognostic biomarkers. The area under curve of the 1-, 3-, and 5-year receiver operating characteristic curves of the training set, test set, and validation set were all >0.6. There were significant differences in the frequency of some gene mutations and scores (immune score, matrix score, and ESTIMATE composite score) between the high- and low-risk groups. Reverse transcription quantitative polymerase chain reaction and Western blot analyses confirmed that CTHRC1, APOD, and S100A12 were significantly upregulated in the tumor group, whereas ASCL2 expression was significantly downregulated. We developed a TME-related gene signature that can predict the prognosis of patients with GC.

## 1. Introduction

Stomach adenocarcinoma (STAD) is one of the most common gastrointestinal malignancies and is characterized by high mortality, malignancy, and strong heterogeneity.^[1]^ The malignant phenotype of STAD depends not only on the intrinsic activity of the cancer cells, but also on the tumor microenvironment (TME) in the recruitment and activation of matrix and immune cell factors.^[2]^ Recent studies have shown that the characteristics of the microenvironment are significantly related to the prognosis and survival of patients with gastric cancer (GC).^[3]^

Tumors represent complex ecosystems, including tumor cells, immune cells, cancer-associated fibroblasts, endothelial cells (EC), parietal cells, additional cells, and more embedded dynamic vascularization of the extracellular matrix.^[4]^ Studies have demonstrated that TME plays an important role in STAD development, prognosis, and immune response.^[5]^ Therefore, we hypothesized that genes associated with the TME can predict the prognosis of patients with GC. Elucidating the characteristics of the TME in STAD will enhance our understanding of its molecular mechanisms and provide a theoretical basis for prognosis and further treatment.

The purpose of this study was to identify the TME features and construct a TME-related prognostic model. We screened 4 key genes as markers of prognostics by applying bioinformatics analysis and constructed a risk-scoring model related to the TME. This model provides a basis and new reference for exploring the molecular mechanisms associated with STAD prognosis and offers a foundation for the treatment of STAD. In addition, we analyzed the mutational signatures and immune infiltration between the high- and low-risk groups.

## 2. Materials and methods

### 2.1. Data acquisition and preprocessing

We downloaded data from The Cancer Genome Atlas (TCGA) database (https://portal.gdc.cancer.gov), including 32 cases of normal tissue samples and 373 cases of cancer tissue sample data and the expression of matrix survival information (Release Date: The 2018-08-23; Release Number: 12.0). After screening samples 11A and 01A (01A represents cancerous tissue, 11A represents normal tissue), 32 normal samples and 373 cancer samples were obtained. Inclusion criteria: the patient was diagnosed with GC by pathological examination; postoperative gastric tissue mRNA examination data were complete. Exclusion criteria: incomplete follow-up data; survival time was 0. Consequently, 348 cancer samples were randomly divided into 2 parts to construct a prognostic model (training set: 244 cases, removing 27 samples without clinical information and 0 survival time; test set: 104 cases). We then downloaded GSE84433 datasets (357 tumor samples) from the Gene Expression Omnibus database (https://www.ncbi.nlm.nih.gov/geo/). After removing 2 cases with a survival time of 0, the prognosis information of 355 tumor samples was used to validate the model. The sequencing platform was a GPL6947 Illumina HumanHT-12 V3.0 expression bead chip.

### 2.2. Cluster analysis

xCELL analysis was performed on the transcriptome data of The Cancer Genome Atlas-Stomach Adenocarcinoma (TCGA-STAD), and the results of 64 cell types were analyzed using univariate Cox analysis to obtain TME-related cells that were significantly related to prognosis. Spearman analysis was used to analyze the correlation among TME-related cells. Simultaneously, the relationship among TME-related cells and their survival rates is shown.

TME patterns were classified according to the fraction of TME-related cells. The cell composition ratio maps in different TME-clusters and the heat maps of cells in different TME-clusters are shown. The survival analysis of different TME-clusters was performed.

### 2.3. Acquisition of candidate genes and hubgenes

The “limma” package was used to analyze differentially expressed genes (DEGs) between the gene expression matrices of different clusters, and the threshold was set as *P* < .05 and |log2FC| > 1. DEGs among different TME-clusters (DEG1+DEG2+ +DEG+ ······ +n) and DEGs between the gastric cancer and the control were used to identify candidate genes. Gene Ontology (GO) and Kyoto Encyclopedia of Genes and Genomes (KEGG) enrichment analyses of candidate genes were performed using the clusterProfiler package.

### 2.4. Construction and validation of prognostic model

A prognostic model was developed by employing univariate Cox analysis, LASSO regression, and multivariate Cox analysis in conjunction with intersection genes. The training, test, and validation sets were stratified into high- and low-risk groups based on the optimal risk score threshold. The survival disparity between these 2 groups was examined using Kaplan–Meier (KM) analysis. Additionally, receiver operating characteristic (ROC) curves of 1-, 3-, and 5-year survival rates were plotted to assess the predictive efficiency of the risk model.

### 2.5. Independent prognostic analysis and correlation analysis of clinical characteristics

Univariate and multivariate Cox regression analyses were performed on 217 TCGA-STAD samples to analyze the independent prognostic value of the risk score. Independent prognostic factors were included in the nomogram, and 1-, 3-, and 5-year calibration curves were drawn.

The correlation between clinical characteristics (such as age, sex, race, TNM stage, stage, grade) and risk score was analyzed. Wilcoxon and Kruskal–Wallis tests were used for the test.

### 2.6. Mutation analysis of high- and low-risk groups

First, basic information on somatic mutations in the high- and low-risk groups was displayed, and the top 10 genes were displayed according to the mutation rate. The mutation annotation format was visualized through the “maftoools” R package to analyze the difference in the mutation frequency of the top 20 mutation genes between the high- and low-risk groups. Next, the frequency distribution of the 96 tumor mutation types in the high-risk and low-risk groups was analyzed. Somatic mutation characteristics in the high- and low-risk groups were detected. The similarity between the mutation characteristics of the high- and low-risk groups and the features included in the COSMIC were analyzed. Finally, GISTIC software (Genomic Identification of Significant Targets in Cancer, developed by the Broad Institute of MIT, Cambridge a nd Harvard, Cambridge) was used to analyze copy number variation (CNV) (such as chromosome arm amplification and deletion) in the high-risk and low-risk groups.

### 2.7. Immune infiltration analysis of high- and low-risk groups

The expression matrix of the training set (244 cases) was used to calculate the proportion of 24 immune cells in the high- and low-risk groups by the single sample gene set enrichment analysis algorithm using the R package “GSVA” and the immune cells with significant differences between the 2 groups were compared (*P* < .05). The differences between the 24 tumor-infiltrating immune cells in the high- and low-risk groups were compared, and the rank-sum test was used to calculate the difference between the 2 groups. A violin plot was drawn using the R package “vioplot.”

### 2.8. Analysis of TME characteristics between high- and low-risk groups

Immune scoring is a standard assay that quantifies the density of immune cells in the TME. It typically involves the quantitative scoring of immune cells (T cells, cytotoxic T cells, etc) in the center of the tumor and at the margins of tumor invasion. Matrix score is a possible way of evaluating certain features or patterns of biological data matrices (gene expression matrices and protein interaction networks). The ESTIMATE composite score is a composite score calculated based on the ESTIMATE algorithm for assessing the content of stromal and immune cells in tumor tissues as well as the purity of the tumor. The ESTIMATE algorithm was used to calculate the infiltration of stromal and immune cells in 244 samples of the high- and low-risk groups in the training set based on gene expression. The rank sum test was used to analyze the differences between the high- and low-risk groups.

### 2.9. Reverse transcription quantitative polymerase chain reaction (RT-qPCR) analysis

In this study, 6 pairs of samples of patients from The Affiliated Hospital of Guizhou Medical University were validated by RT-qPCR and Western blotting, including 6 precancerous and 6 tumor tissues. The tissues were isolated and immediately stored in liquid nitrogen for RT-qPCR analysis. Total RNA was extracted using a Total RNA Extraction Kit (Solarbio, Beijing, China). RNA concentration was spectrophotometrically quantified using a NanoDrop spectrophotometer (Thermo, Waltham). Next, reverse transcription of mRNA was performed using EasyScript One-Step gDNA Removal and cDNA Synthesis SuperMix (Transgen, Beijing, China). mRNA expression was analyzed on a CFX96 Touch thermocycler (Bio-Rad, Hercules) using TransStart Green qPCR SuperMix UDG (Transgen, Beijing, China). The amount of mRNA was normalized to that of actin, using the 2^−ΔΔCT^ method. Primers used are listed in Table S1, Supplemental Digital Content, https://links.lww.com/MD/P789. This study was approved by the Ethics Committee of the Affiliated Hospital of Guizhou Medical University (Ethics Approval No. 2020252).

### 2.10. Western blot analysis

The tissue was homogenized using a grinding machine (Jingxin, Shanghai, China) with radioimmunoprecipitation buffer (high) (Solarbio) containing phenylmethylsulfonyl fluoride (Beyotime, Shanghai, China). The machine was precooled to −30°C, and tissues were homogenized for 10 seconds each time (3 times in total with an interval of 10 seconds). After measuring the protein concentration, 1/3 volume of 5× protein loading buffer was added and heated at 95°C for 15 minutes. Sodium dodecyl sulfate polyacrylamide gel electrophoresis was used to separate the proteins, and a pageRuler prestained protein ladder was used for protein molecular weight estimation. After separation, the protein was transferred onto a 0.22 μm PVDF membrane. The membranes were blocked by incubation in 5% skim milk for 2 hours at room temperature. Membranes were then placed in a primary antibody buffer and incubated overnight at 4°C. The membranes were blocked with the corresponding secondary antibody for 2 hours at room temperature. Finally, the gel imaging system was used for analysis (Bio-Rad). The antibodies used in this study are listed in Table S2, Supplemental Digital Content, https://links.lww.com/MD/P789.

### 2.11. Statistical analysis

Data were analyzed using R software (version 4.2.3; The R Foundation for Statistical Computing, Vienna, Austria). The mRNA expression levels of the genes and multivariate Cox coefficients were used to construct a risk score. The hazard ratio in the forest plot was determined using univariate and multivariate Cox regression analyses. KM curves and log-rank tests were used for survival analysis. Differences between the groups were assessed using paired *t*-tests. Statistical significance was set at *P* < .05.

## 3. Results

### 3.1. X-cell and NMF clustering analysis

**Figure 1.**
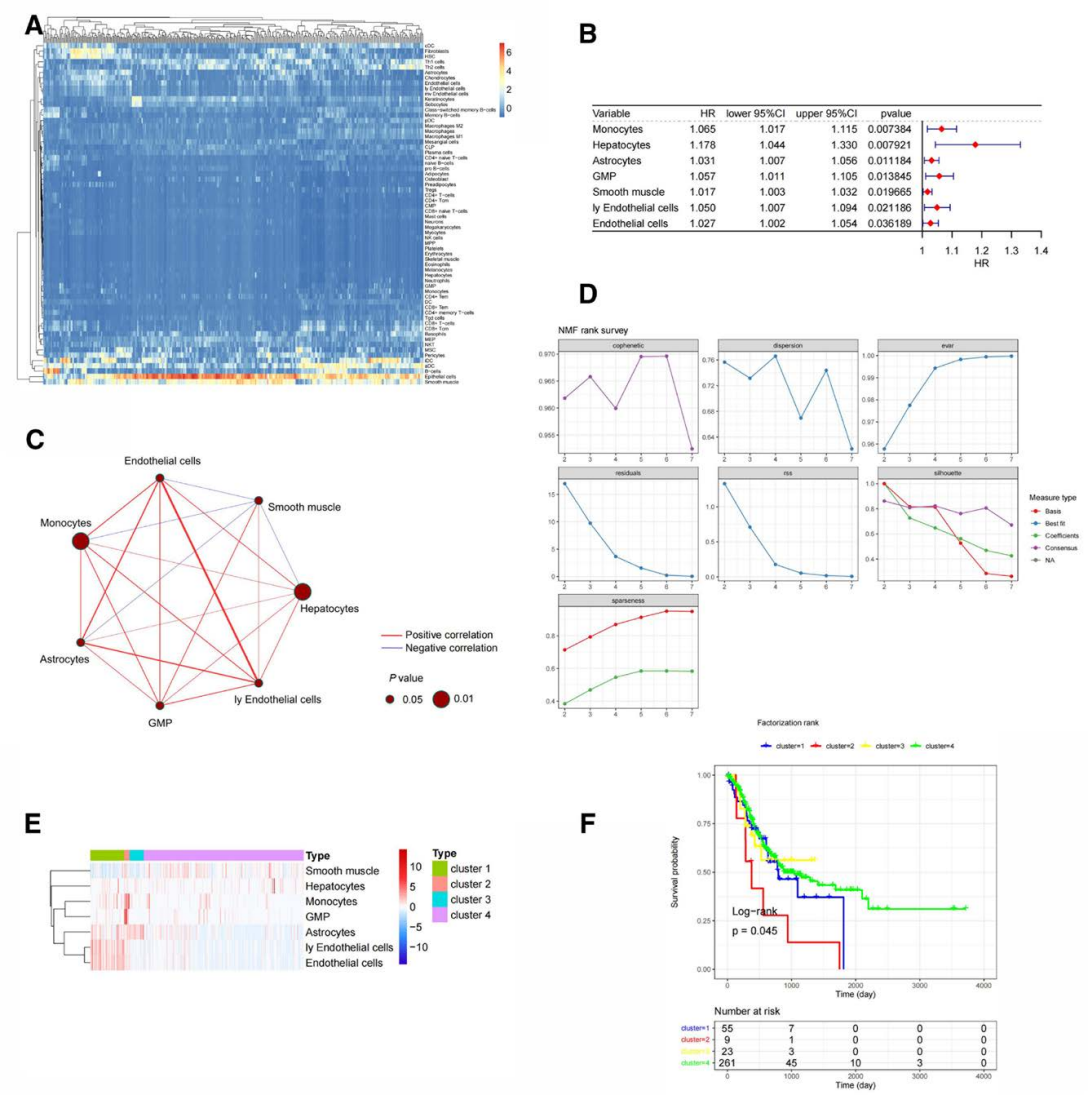
(A) Heat map of the 64 TME-relatived cell. (B) Forest map of the 7 significantly different TME-relatived cell. (C) Correlation network map of the 7 significantly different TME-relatived cell. (D) NMF clustering of the 7 significantly different TME-relatived cell. (E) Heat maps of the 7 significantly different TME-relatived cell in the different clusters. (F) Survival analysis of the different clusters. CI = confidence interval, HR = hazard ratio, TME = tumor microenvironment.

First, the rank values of 64 TME-related cells were calculated for 373 cancer samples (Fig. 1A). Univariate Cox regression analysis was performed on 64 TME-associated cells to identify TME-phase cells that were significantly associated with prognosis (Fig. 1B). Among these cells, astrocytes, EC, granulocyte-monocyte progenitors, hepatocytes, lymphatic EC, monocytes, and smooth muscle cells are TME-related cells that are significantly related to prognosis. We analyzed the correlation between 7 TME-related cells with *P* < .05 Spearman. At the same time, we explore the relationship between TME cells and their survival rate (Fig. 1C). The R package NMF (version 0.23.0) was used to classify the 7 prognostic significant TME-related cell rank values of the 373 tumor samples, and *k* = 4 was selected. The 4 clusters were cluster 1, cluster 2, cluster 3, and cluster 4 (Fig. 1D). Heat maps of TME-related cells in different TME-clusters are displayed (Fig. 1E). Survival analysis was performed for different clusters (Fig. 1F). Survival analyses between different clusters showed *P* < .05, indicating a significant difference.

### 3.2. Differential gene analysis and hubgene functional enrichment analysis

**Figure 2.**
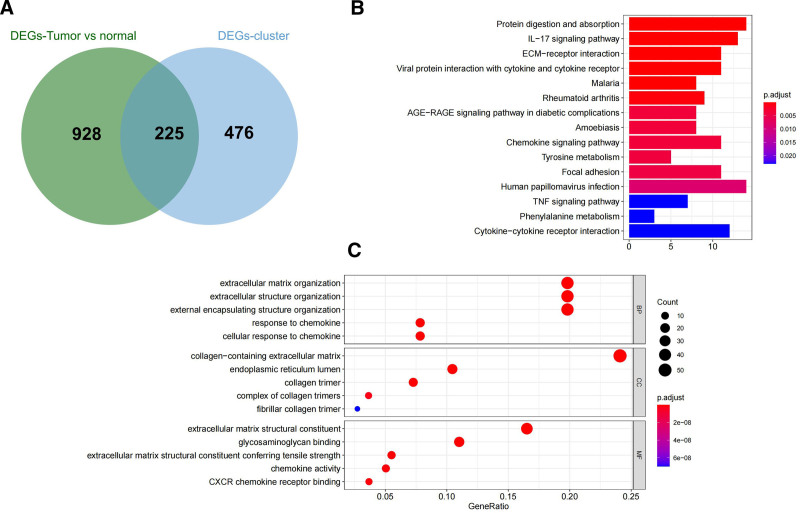
(A) Venn diagram of DEGs (tumor vs normal) versus DEGs (between 4 clusters). (B) Enriched GO terms. (C) Enriched KEGG pathways. (The size of the ball represents the number of enriched genes, the color represents the adjusted value, and the vertical axis represents KEGG pathways and GO terms.) DEGs = differentially expressed genes, GO = Gene Ontology, KEGG = Kyoto Encyclopedia of Genes and Genomes.

In the comparison of clusters 2 and 1, there were 108 differential genes (88 upregulated and 20 downregulated). The number was 118 (60 upregulated genes and 58 downregulated), 140 (1 upregulated gene and 139 downregulated), 227 (100 upregulated and 127 downregulated), 354 (12 upregulated and 342 downregulated), 146 (35 upregulated and 111 downregulated) when it came to cluster 3 versus cluster 1, cluster 4 versus cluster 1, cluster 3 versus cluster 2, cluster 4 versus cluster 2, cluster 4 versus cluster 3, respectively. In the comparison of tumor (373 samples) versus normal (32 samples) samples, 1153 significantly different genes were detected, with 721 upregulated genes and 432 downregulated genes. The union of DEGs across the 4 clusters resulted in 701 DEGs (DEGs-cluster). By intersecting 1153 differential genes between disease and normal samples (DEGs-TumorvsNormal), we obtained 225 intersecting genes (Fig. 2A).

Based on enrichment analysis of GO and KEGG pathways, the common functions and related pathways of a large number of genes in the intersection gene set were identified. A total of 362 GO terms were obtained, including 278 biological process terms (response to chemokines, chemokine-mediated signaling pathway, myeloid leukocyte migration, leukocyte chemotaxis, etc have been obtained), 58 molecular functions (chemokine activity, CXCR chemokine receptor binding, chemokine receptor binding, heparin-binding, etc), and 26 molecular function cellular component terms (containing collagen extracellular matrix, collagen trimer, endoplasmic reticulum lumen, collagen trimer complex, etc); 20 KEGG pathway (IL-17-receptor signaling pathways, the extracellular matrix, viral protein interaction, the interaction of cytokines and cytokine receptors, the AGE-RAGE signaling pathways in the role of diabetes complications, etc) were enriched (Fig. 2B and C).

### 3.3. Construction and validation of prognostic models

**Figure 3.**
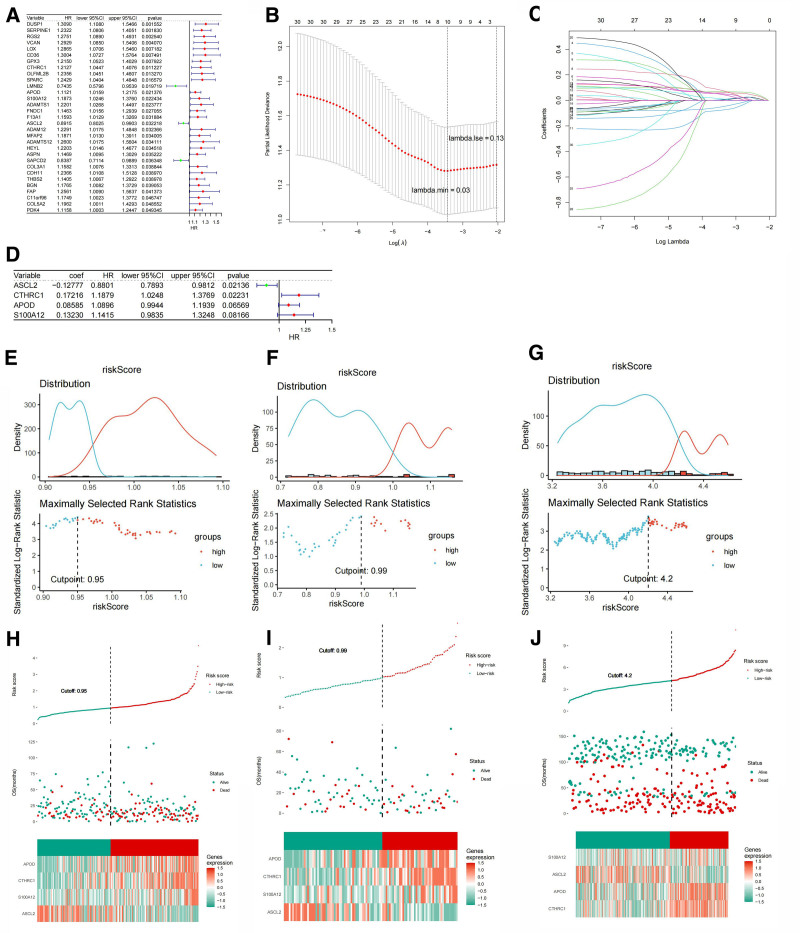
(A) Univariate forest plot of the association between hubgene expression and overall survival in STAD patients. (B) Partial likelihood deviation plot. (C) Graph of gene coefficients. (D) Multivariate forest plot of the association between lassogene expression and overall survival in STAD patients. (E) Best threshold values of the training set. (F) Best threshold values of the test set. (G) Best threshold values of the external validation set. Risk curves, scatter plots, and model gene expression heatmaps of high- and low-risk groups of patients in the (H) training set, (I) test set, and (J) external validation set. STAD = stomach adenocarcinoma.

According to the ratio of 7:3, 348 patients were randomly divided into the training set (244 cases; the sample size was calculated based on the principle of 10–20 EPV) and the internal validation set (104 cases). Thirty-one prognosis-related genes were identified in the training set; 31 prognostic related genes were obtained, which were S100A12, CTHRC1, MFAP2, F13A1, PDK4, ASCL2, ADAMTS1, C11orf96, HEYL, SERPINE1, DUSP1, BGN, COL3A1, CD36, LMNB2, SAPCD2, ADAM12, FAP, SPARC, LOX, VCAN, COL5A2, FNDC1, ADAMTS12, OLFML2B, CDH11, RGS2, GPX3, THBS2, APOD, and ASPN. The R package “forestplot” was used to draw a univariate Cox forest plot, where the outcome event studied was death (Fig. 3A).

The 31 prognostic genes obtained from the previous univariate Cox analysis were selected in the training set using the R package “glmnet” using the LASSO algorithm. LASSO logistic regression was implemented to select the strongly correlated features. Figure 3B was the partial likelihood deviation plot, and Figure 3C was the graph of gene coefficients. According to the figure, the partial likelihood deviation was the smallest when lambda.min was 0.03, and the corresponding best *λ* value was 10, that is, 10 feature genes were screened: DUSP1, SERPINE1, RGS2, VCAN, CD36, CTHRC1, LMNB2, APOD, S100A12, and ASCL2.

Through multivariate Cox regression analysis in the training set, using 10 prognosis-related genes identified from the LASSO analysis, we ultimately identified 4 genes (CTHRC1, APOD, S100A12, and ASCL2) as prognostic biomarkers for constructing the risk model (Fig. 3D). To evaluate the prognostic value of the risk model, STAD patients were scored based on the expression levels of the 4 model genes and the risk coefficient obtained by multivariate Cox regression. The risk score was calculated using the following formula: risk score = 0.172163254 × CTHRC1 + 0.085845462 × APOD + (−0.127769086)  × ASCL2 + 0.132302193 × S100A12. The surv_cutpoint function of the R package “survminer” was used to screen the optimal thresholds for the training and test sets. Samples above the optimal threshold were classified as the high-risk group and those below the optimal threshold were classified as the low-risk group. The best threshold value of the training set (Fig. 3E) was 0.95, the best threshold value of the test set (Fig. 3F) was 0.99, and the best threshold value of the external validation set (Fig. 3G) was 4.2. Risk curves were drawn according to the risk scores (Fig. 3H–J). The risk curve is composed of 3 parts: the upper, middle, and lower parts. The abscissa was the patient sample ranked according to the risk score. The top graph shows the ordinate risk score and survival time, with a dotted line indicating the optimal threshold value for the risk score. The bottom graph displays a heatmap of the expression levels of the model genes in the high- and low-risk groups.

**Figure 4.**
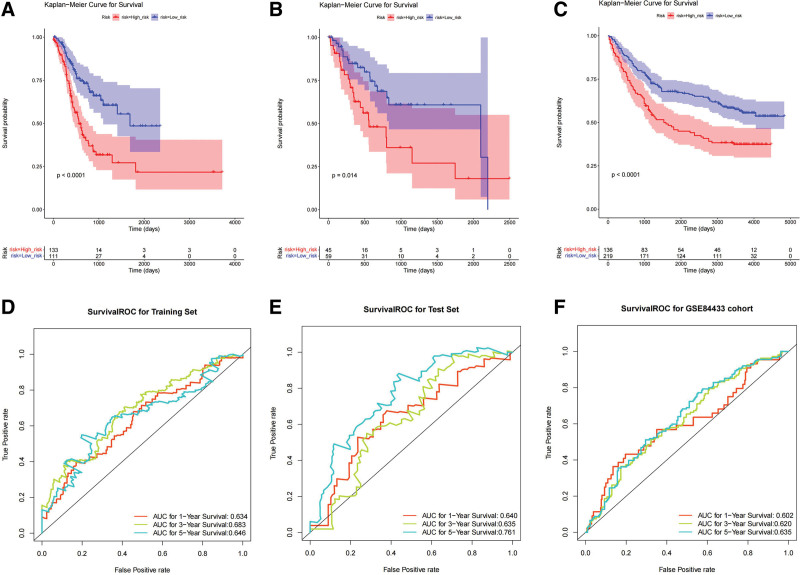
Kaplan–Meier survival analysis of the (A) training set, (B) test set, and (C) validation set. ROC curves of the (D) training set, (E) test set, and (F) validation set. ROC = receiver operating characteristic.

To further confirm the effect of the model genes on the survival prognosis of patients, prognostic survival analysis was performed between the high- and low-risk groups. The R package “survival” was used to calculate the survival analysis, and the “survminer” package was used to summarize and visualize the results of the survival analysis KM survival curves (Fig. 4A–C). Patients in the high-risk group had shorter survival times than those in the low-risk group did. To further evaluate the effectiveness of the risk model, ROC was used to calculate the area under the curve (AUC) of the model to evaluate the effectiveness of the model. According to the STAD risk model obtained by univariate Cox and multivariate Cox regression analysis, the R package “pROC” was used to draw ROC curves with 1, 3, and 5 years as survival time nodes, and the AUC of the 1-, 3-, and 5-year ROC curves of the training, test, and validation sets were all >0.6, indicating that the performance of the risk model was good (Fig. 4D–F).

### 3.4. Construction of nomogram and analysis of its correlation with clinical features

In this study, univariate and multivariate Cox analyses based on risk score and clinicopathological features were performed in the training set (217 cancer samples) using the R package “survminer” to investigate whether risk characteristics were independent risk factors for STAD survival.

**Figure 5.**
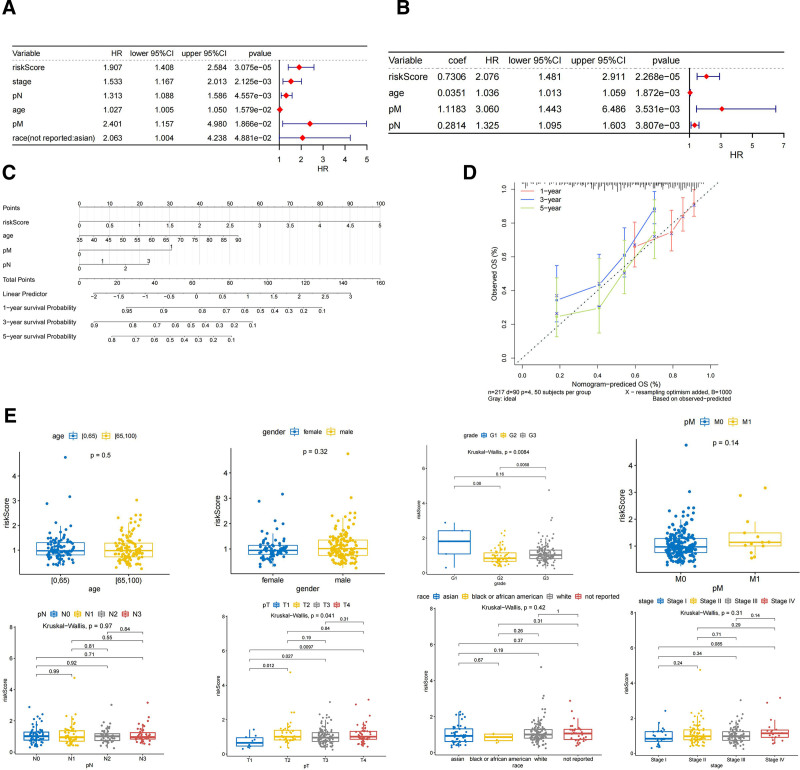
Forest plots of (A) univariate and (B) multivariate Cox in TCGA-STAD. (C) Nomogram and a calibration curve of an independent prognostic model. (D) Calibration curve of an independent prognostic model. (E) Analysis of risk scores according to clinicopathological features. TCGA-STAD = The Cancer Genome Atlas-Stomach Adenocarcinoma.

Forest plots of univariate and multivariate Cox analyses were drawn using the R package “forestplot” where the outcome event studied was death (Fig. 5A and B). The risk score, age, M stage, and N stage were independent prognostic factors for STAD in TCGA-STAD.

Based on the independent prognostic factors screened by TCGA multivariate Cox independent prognosis, we used the “RMS” package to construct the independent prognostic model of clinical factors, risk score, age, M stage, and N stage. Then drew the nomogram and predicted the possible 1-, 3-, and 5-year survival rate of STAD patients. The C-index of the nomogram was 0.6617 (Fig. 5C). We constructed an overall calibration curve to verify the validity of the nomogram. The distribution of the calibration curve was close to the diagonal line, indicating a better prediction effect of the model (Fig. 5D).

The correlation between clinical characteristics (such as age, sex, grade, race, stage, TNM stage) and the risk score was calculated in the training set (217 cancer samples). The test methods used were as follows: Wilcoxon (2 groups) and Kruskal–Wallis (3 groups or more), where grade (*P* = .0084) and T stage (*P* = .041) were significantly correlated with the risk scores. To some extent, the risk score was affected by grade and T stage (Fig. 5E).

### 3.5. Genetic characteristics are different between the 2 groups

**Figure 6.**
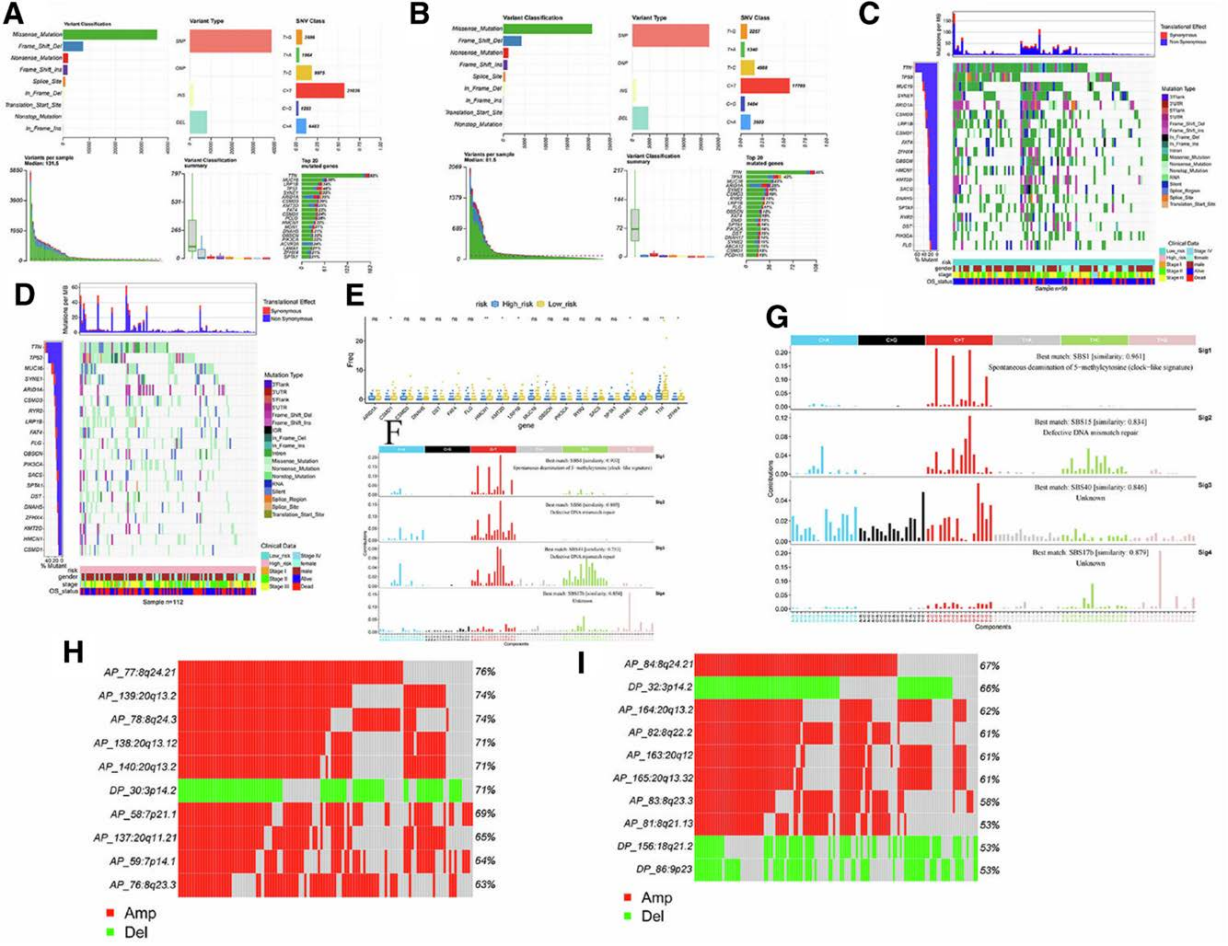
(A and B) Basic information on somatic mutations in patients in the high- and low-risk groups. (C and D) Distribution and phenotype of genes with higher proportions of mutations in each of the 20 high- and low-risk groups. (E) The difference in mutation frequency of 20 genes with higher mutation proportion in the high- and low-risk groups between the high- and low-risk groups. (F and G) Similarity between the mutational signatures of the high- and low-risk groups and the signatures in COSMIC. (H and I) CNV profiles in the high- and low-risk groups. CNV = copy number variation.

We display mutation information in these samples (high-risk group samples: 130; low-risk group samples: 111), showing the top 20 genes according to the mutation rate, visualizing in mutation annotation format by the “maftoools” R package. The variant classification, variant type, single nucleotide variant category, variant in each sample, variant classification summary, and top 20 mutant genes are shown respectively (Fig. 6A and B). Statistical analysis was performed on the mutation data of the high-risk and low-risk groups (high-risk group samples: 112 cases; low-risk group samples: 99 cases), showing the distribution and phenotype of 20 genes with high mutation proportions in the 2 groups. Among them, more than 40% of the sample in had TTN and TP53 mutations (Fig. 6C and D). The mutation frequencies of these 20 genes were further analyzed in the high- and low-risk groups (Fig. 6E). The mutation frequencies of CSMD1, HMCN1, KMT2D, LRP1B, SYNE1, TTN, and ZFHX4 were significantly different between the high- and low-risk groups.

To determine the relationship between the mutation frequency distribution of STAD tumor samples and the features included in COSMIC, the somatic mutation signatures of the high- and low-risk groups were detected separately, and the similarity between the 4 mutation signatures of the high/low risk groups and the mutation signatures included in COSMIC were analyzed, and the results in the 2 groups are shown. The different mutation characteristics of the high-risk group were mainly related to the spontaneous deamination of 5-methylcytosine and DNA mismatch repair defects. The different mutation characteristics of the low-risk group were mainly related to the spontaneous deamination of 5-methylcytosine and DNA mismatch repair defects (Fig. 6F and G).

GISTIC software was used to analyze the CNV (chromosome arm amplification and deletion) of the high- and low-risk groups, showing the most frequently altered (amplification or deletion) CNVs ranked according to frequency. Each column represents one sample, and each row represents one CNV fragment (Fig. 6H and I).

### 3.6. The immune cells are significantly different between the 2 groups

**Figure 7.**
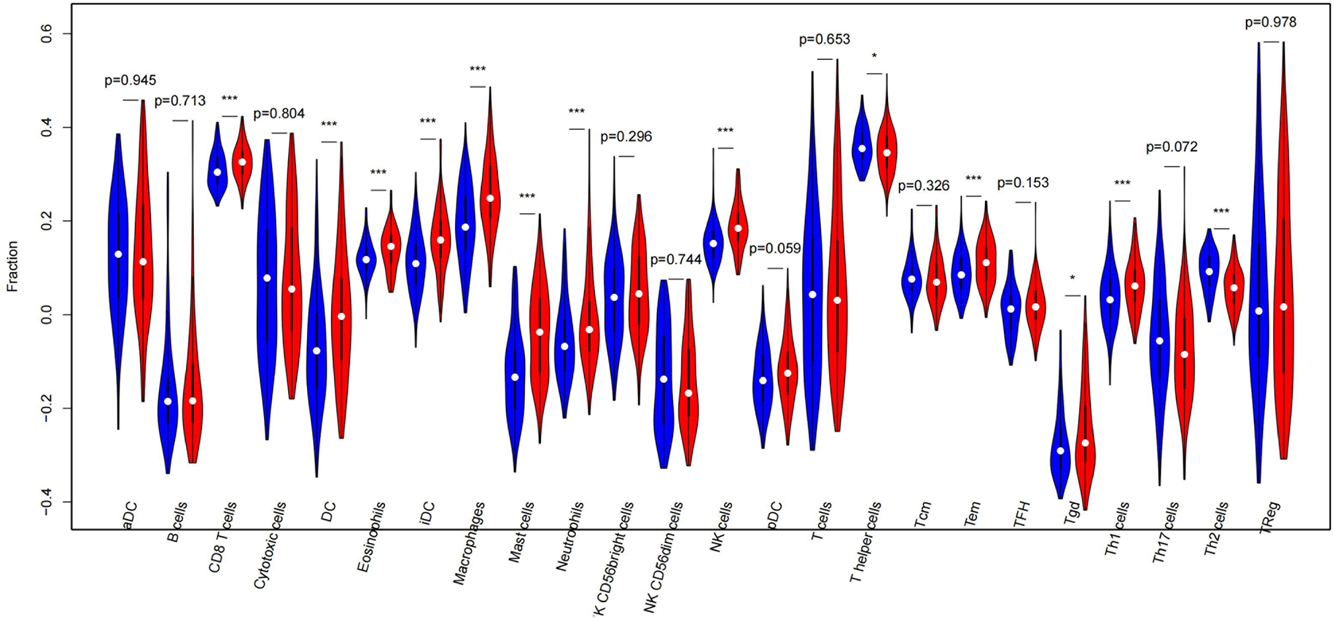
Differences of the 24 TIICs. TIICs = tumor-infiltrating immune cells

The expression matrix of the training set (244 cases) was used to calculate the proportion of 24 immune cells in the high- and low-risk groups by the single sample gene set enrichment analysis algorithm using the R package “GSVA.” Immune cells with significant differences between the 2 groups were compared (*P* < .05). The differences between the 24 tumor-infiltrating immune cells in the high- and low-risk groups were compared, and the rank-sum test was used to calculate the difference between the 2 groups. The violin plot was drawn by the R package “vioplot.” Among them, there were 13 immune cells with significant differences (*P* < .05): CD8 T cells, DC, eosinophils, iDC, macrophages, mast cells, neutrophils, NK cells, T helper cells, Tem, Tgd, Th1 cells, and Th2 cells (*P < .05, **P < .01, ***P < .001, ****P < .0001) (Fig. 7).

### 3.7. The TME-related scores were higher in the high-risk group

**Figure 8.**
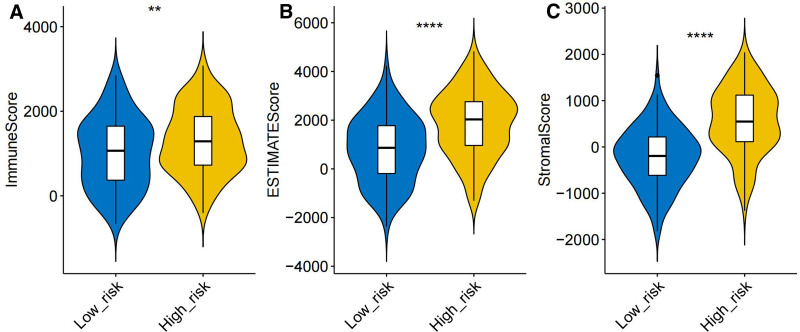
(A) Immune scores. (B) ESTIMATE composite scores. (C) Matrix scores.

We used the ESTIMATE algorithm to calculate the infiltration of stromal and immune cells in 244 samples of the high- and low-risk groups in the training set based on gene expression. The final samples obtained 3 scores: the matrix score, immune score, and ESTIMATE composite score. These scores were compared between the high- and low-risk groups using the rank-sum test. The immune score (Fig. 8A), ESTIMATE composite score (Fig. 8B), and matrix score (Fig. 8C) were significantly higher in the high-risk group than in the low-risk group.

### 3.8. Expression levels of key genes are consistent with the risk model

**Figure 9.**
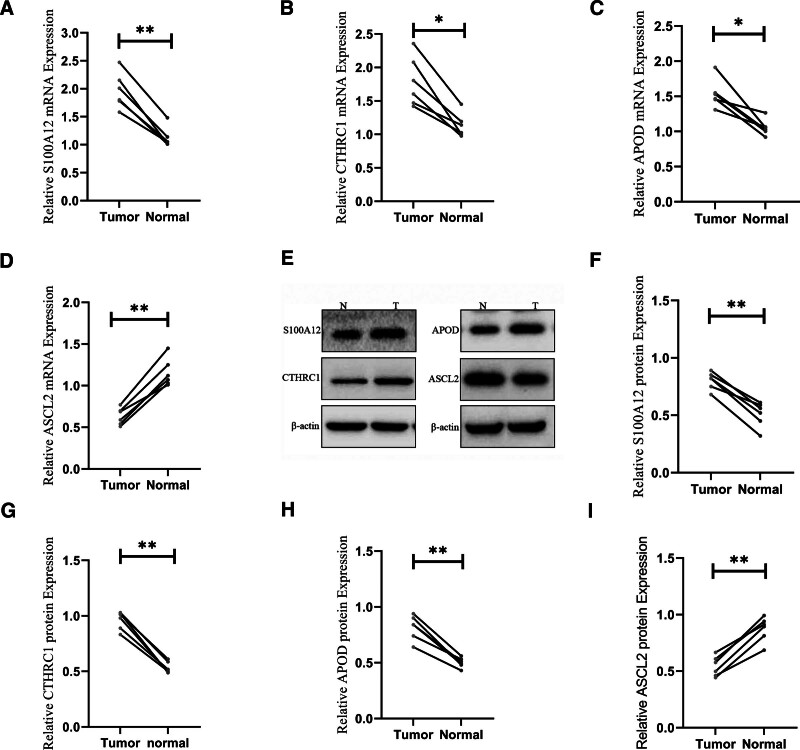
(A–D) Relative S100A12, CTHRC1, APOD and ASCL2 mRNA level. (E–I) Relative S100A12, CTHRC1, APOD, and ASCL2 protein level. Statistical significance is marked by asterisks * *P* < .05 compared to the control group. ** *P* < .01 compared to the control group.

In this study, the expression levels of key genes were verified using RT-qPCR and Western blotting. The results showed that the expression levels of CTHRC1, APOD, and S100A12 in the tumor group were significantly upregulated, whereas the expression levels of ASCL2 in the tumor group were significantly downregulated (Fig. 9A–I).

## 4. Discussion

In our study, by analyzing the potential factors affecting the prognosis of GC in the TME, a risk signature was identified in different prognostic subgroups of GC. Four predictors (CTHRC1, APOD, S100A12, and ASCL2) were selected as prognostic biomarkers to construct the risk model. The 1-, 3-, and 5-year AUCs of the model in the training set were 0.634, 0.683, and 0.646, respectively, indicating good predictive values. In the internal and external validation sets, the 1-, 3-, and 5-year AUCs were 0.640, 0.635, 0.761, and 0.602, 0.620, 0.635, respectively, which can accurately predict the prognosis. The training set samples were then divided into high- and low-risk groups according to the risk score, which was used as an independent prognostic factor to predict the prognosis of GC combined with age, M stage, N stage, and other indicators. A nomogram was drawn to predict the 1-, 3-, and 5-year survival rates of patients with GC. The C-index of the nomogram is 0.6617. A calibration curve with a diagonal results in a better prediction accuracy. Finally, the differences in mutation and TME characteristics between the high- and low-risk groups were analyzed again to reveal the genetic and environmental factors affecting the prognosis of GC. The gene functions and pathways of prognostic factors in the model suggest that these factors jointly promote the formation of a repressive immune microenvironment from the aspects of chemokines and immunosuppression ultimately promote tumor progression and metastasis.^[6]^

Among the 4 predictors, collagen triple helix repeat containing 1 (CTHRC1) had the greatest impact on prognosis. CTHRC1 is a highly conserved secretory glycoprotein with a secretory function and is mainly distributed in the cytoplasm and intercellular space. In recent years, it has been found that CTHRC1 is abnormally expressed in various solid tumors and is closely related to tumor invasion, metastasis, and survival prognosis.^[7]^ Some studies suggest that CTHRC1 creates conditions for the occurrence and development of carcinogenesis by reducing synthesis of the extracellular matrix.^[8]^ At the same time, CTHRC1 belongs to a class of TGF-β-sensitive receptor inhibitors, and CTHRC1 ligands combine with the corresponding type I and type II receptors to produce signals. This signaling pathway can be transmitted to Smad proteins through TGF-β family members, followed by phosphorylation and translocation to the nuclear environment to play a regulatory role.^[9]^ In addition, it can affect the differentiation and intimal hyperplasia of muscle cells. In conclusion, abnormal expression of CTHRC1 is observed in a variety of human solid tumors and may be closely related to the malignancy and invasion of cancer tissues. However, the underlying mechanism requires further investigation.

S100A12 belongs to the S100 protein family. They play important roles in cell proliferation, differentiation, muscle contraction, gene expression, secretion, and apoptosis through calcium signal transduction, influencing hormone secretion inhibiting tubulin assembly, and inhibiting protein kinase C-mediated phosphorylation. Dysfunction can lead to various diseases.^[10]^ The gene of human S100 is located in the 1q21 region of the chromosome. This region is frequently rearranged in tumor tissues, resulting in uncontrolled S100 gene expression. Due to the above characteristics of S100, it has been found in recent years that the abnormal expression of S100 in tumor tissues, especially in tumor tissues of neuroectodermal origin, is correlated with the stage and prognosis of the disease in recent years.^[11]^ Bioinformatics analysis has revealed that TNF and S100A11/A12/A13 are dysfunctional in a variety of diseases.^[12]^ It has been found that S100A12 is upregulated in glioma tissues and its expression is correlated with WHO grade and tumor size.^[12]^ Researchers have further confirmed that S100A12 knockout could inhibit the proliferation, migration, and metastasis of glioma cells by regulating cell apoptosis and epithelial-mesenchymal transition .^[13]^ Another study has suggested that S100A12 plays an important role in resisting microbial infection and maintaining immune homeostasis.^[14]^ Additionally, research has shown that in pharyngeal carcinoma, immune activation is associated with strong S100A12.^[15]^ Another investigation on PTC confirmed that S100A12 was significantly upregulated in thyroid cancer; S100A12 silencing significantly inhibited the proliferation, migration, and invasion of PTC. Knockout of S100A12 significantly reduces the expression of CyclinD1, CDK4, and p-ERK in PTC cells and results in G0/G1 phase arrest by inhibiting the ERK signaling pathway.^[16]^

Apolipoprotein D (APOD) is a member of the apolipoprotein superfamily. The well-known biological functions of APOD are mainly related to lipid metabolism and neuroprotection.^[17]^ One study found that APOD in Th17 cells have higher mRNA and protein levels than in Th1, Th2 subtypes, and Treg cells, and the gene encoding protein and Th17 cell function are closely related to these cells producing inflammation, which is associated with hardening and angiogenesis. Researchers further found that the high expression of this gene in Th17 cells is related to the acetylation of H2BK12 in its promoter.^[18]^ Another study suggested that APOD expression can independently predict the prognosis of breast cancer.^[19]^

ASCL2 (Achasutelike2) is an E protein. It can increase the activation of T cell surface antigen expression of CXCR5 and regulate T cell homing to follicles to initiate Tfh cell development. Researchers^[20]^ found that ASCL2 transcription factor expression was specifically upregulated in germinal center B cells. Forced expression of ASCL2, when overexpressed by retroviruses in B cells in vivo and in vitro, was found to promote GC B cell development, while enhancing antibody production and affinity maturation. In contrast, B cells in the germinal center reaction ASCL2 defects will lead to damage, including lower GC B cells and antibody affinity. These results suggest that ASCL2 is a key regulator of humoral immunity, particularly in the differentiation of GC B cells. Genome-wide analysis revealed that ASCL2 directly regulates several GC B cell-related genes, including AID; overexpression of AID in ASCL2-deficient B cells rescued the defect in antibody production. In a study of colon cancer, researchers found that another option to inhibit ASCL2 expression was promoter methylation mediated by TET2-BCLAF1.^[21]^ Another study on colon cancer suggested that the dynamics of activated enhancers inhibit CRC progression and that patient-specific enhancer patterns are affected by precision therapy.^[22]^ Other colon cancer studies have suggested a link between the dysregulation of ASCL2 and MSS in CRC.^[23]^

The expression levels of the 4 prognostic genes from TCGA were validated using the GSE84433 dataset, and the results showed consistent expression patterns between the 2 datasets. RT-qPCR and Western blot analyses confirmed that CTHRC1, APOD, and S100A12 expression was significantly upregulated in the tumor group, while ASCL2 expression was significantly downregulated.

To further explore the genetic factors between the high- and low-risk groups, a correlation analysis was performed. The results showed that somatic mutations were associated with spontaneous deamination of 5-methylcytosine and DNA mismatch repair. The above phenomena confirm that the intrinsic characteristics of tumor cells, including genetic changes, epigenetic changes, metabolic reprogramming, and signal release, are the key determinants of TME shaping.^[24]^ Changes in the intrinsic characteristics of cancer cells can alter secreted substances, cell surface receptors, or ligands; affect the cargo load and abundance of extracellular vesicles ; and alter the use of nutrients, leading to a wide range of changes in the tumor immune environment.

By analyzing immune infiltration between the high- and low-risk groups, significant differences were found in 13 of the 24 immune cells. This is consistent with the results of previous studies by Liu et al,^[25]^ Chen et al,^[26]^ and others. There have been a lot of studies demonstrating that the immune response throughout the whole process of tumor development and the immune mechanism in tumorigenesis plays an extremely important role in the process.

To study the effect of the TME on prognosis, we analyzed the immune, matrix, and ESTIMATE scores of the high- and low-risk groups. This phenomenon suggests the influence of the TME on tumor prognosis. Invasive stromal and immune cells within the TME constitute a significant portion of nonmalignant cells in tumor tissues. They interfere with molecular tumor signaling studies and play a crucial role in tumor biology.^[25]^

A typical example is GC, which includes hepatoid adenocarcinoma of the stomach (HAS). HAS is a special subtype of GC, which is mainly characterized by the presence of hepatocyte-like differentiation regions in tumor tissues and the production of alpha-fetoprotein. HAS has highly malignant biological behavior and is prone to lymph node metastasis and liver metastasis, accounting for 0.3% to 1% of all GC.^[27]^ HAS is essentially a GC, but its histomorphology is similar to that of hepatocellular carcinoma. It highly expresses alpha-fetoprotein, GPC3, SALL4 genes, and other liver cancer-related molecular markers.^[28]^ It can also be seen from the X-cell analysis in this study that the size of the samples with hepatocyte markers was very small, while the survival analysis showed that the prognosis of these samples with hepatocyte gene markers was extremely poor. Kang et al demonstrated the potential of the immune prognostic index as a reliable indicator for predicting the prognosis of HAS. Immune checkpoint inhibitors combined with chemotherapy appear to function well in the clinical practice of HAS. A portion of HAS patients with TP53 status may be a plausible explanation for this phenomenon, and these patients are likely to exhibit a good response to ICIs therapy.^[29]^ This result is consistent with the conclusions of the research team at Kyushu University in Japan.^[30]^ They evaluated the proportion of liver-like components in tumors and suggested that HC content is an independent prognostic factor for HAS.

Therefore, it is necessary to understand the characteristics of the TME as a whole and to consider multiple levels of complexity in prognostic evaluation and treatment.^[31]^ Treatment interventions, such as chemotherapy and immunotherapy, may affect the TME; in turn, they are affected by patients with TME and systemic changes.^[32]^ In addition to chemotherapy and immunotherapy, radiotherapy is a widely used treatment modality for tumors, with extensive applications in the medical field. Studies have demonstrated that radiotherapy not only eradicates tumor cells but also induces the release of pro-inflammatory molecules and infiltration of immune cells, thereby altering the TME.^[33]^ Furthermore, research indicates that the response of cancer cells to radiation is modulated by the TME.^[34]^ In conclusion, radiotherapy can affect the TME. Comprehensive consideration of a reasonable combination of these factors is essential for accurate prognosis assessment and further development of reasonable treatment strategies.

In summary, this study has the following advantages. First, we used multiple advanced machine learning methods to distinguish different prognostic subgroups of GC and then screened out representative candidate prognostic genes. In addition, this is a prognostic model of GC based on TME and is based on the close relationship between TME and the prognosis of GC. The genes involved represent comprehensive and main aspects and provide a more accurate method than other models. The new model aims to identify key genes associated with TME through bioinformatic analysis, while the prognostic method was designed to further validate the correlation between clinical features, such as TNM staging and risk scores. The strength of the new model lies in its capacity to accurately select key genes, account for the combined effects of various biomarkers, and provide comprehensive prognostic information, thereby offering precise theoretical guidance for future research. Generally, bioinformatics-based models yield more accurate prognostic predictions because they consider a broader range of biomarkers and molecular characteristics. Finally, modulating the genes contained in the model may be a potential therapeutic strategy and provide a new approach for improving the prognosis of patients with GC.

Our study had some limitations. First, the data from this study were retrospective, and prospective studies are needed to clarify this issue. Second, the data from different platforms may be biased. Third, more samples are needed for experimental verification of the results to be more convincing. In the future, we plan to extensively validate the constructed model through animal experiments to confirm our conclusions.

## 5. Conclusion

We constructed and verified a TME-related signature for GC prognosis of GC. It can effectively predict the prognosis of GC. Targeted therapy for TME-related cells, biological processes, and signaling pathways is a promising therapeutic strategy.

## Author contributions

**Conceptualization:** Pailan Peng.

**Data curation:** Xuelian Li.

**Formal analysis:** Fanghui Zhou.

**Methodology:** Yi Xie.

**Project administration:** Pailan Peng.

**Software:** Yi Xie.

**Validation:** Xuelian Li, Pailan Peng.

**Writing – original draft:** Xuelian Li.

**Writing – review & editing:** Pailan Peng.

## Supplementary Material



## References

[1] Ren N Liang B Li Y. Identification of prognosis-related genes in the tumor microenvironment of stomach adenocarcinoma by TCGA and GEO datasets. Biosci Rep. 2020;40:BSR20200980. 33015704 10.1042/BSR20200980 PMC7560520

[2] Kumar V Ramnarayanan K Sundar R. Single-cell atlas of lineage states, tumor microenvironment, and subtype-specific expression programs in gastric cancer. Cancer Discov. 2022;12:670–91. 34642171 10.1158/2159-8290.CD-21-0683 PMC9394383

[3] Oya Y Hayakawa Y Koike K. Tumor microenvironment in gastric cancers. Cancer Sci. 2020;111:2696–707. 32519436 10.1111/cas.14521 PMC7419059

[4] de Visser KE Joyce JA. The evolving tumor microenvironment: from cancer initiation to metastatic outgrowth. Cancer Cell. 2023;41:374–403. 36917948 10.1016/j.ccell.2023.02.016

[5] Zeng D Zhou R Yu Y. Gene expression profiles for a prognostic immunoscore in gastric cancer. Br J Surg. 2018;105:1338–48. 29691839 10.1002/bjs.10871 PMC6099214

[6] Kang B Camps J Fan B. Parallel single-cell and bulk transcriptome analyses reveal key features of the gastric tumor microenvironment. Genome Biol. 2022;23:265. 36550535 10.1186/s13059-022-02828-2 PMC9773611

[7] Sial N Ahmad M Hussain MS. CTHRC1 expression is a novel shared diagnostic and prognostic biomarker of survival in six different human cancer subtypes. Sci Rep. 2021;11:19873. 34615943 10.1038/s41598-021-99321-w PMC8494806

[8] Toomey BH Mitrovic SA Lindner-Liaw M. Activated CTHRC1 promotes glycolysis in endothelial cells: implications for metabolism and angiogenesis. Vascul Pharmacol. 2023;153:107246. 38040222 10.1016/j.vph.2023.107246 PMC10733615

[9] Mei D Zhu Y Zhang L Wei W. The role of CTHRC1 in regulation of multiple signaling and tumor progression and metastasis. Mediators Inflamm. 2020;2020:9578701. 32848510 10.1155/2020/9578701 PMC7441421

[10] Gonzalez LL Garrie K Turner MD. Role of S100 proteins in health and disease. Biochim Biophys Acta Mol Cell Res. 2020;1867:118677. 32057918 10.1016/j.bbamcr.2020.118677

[11] Peris K Fargnoli MC Kaufmann R. European consensus-based interdisciplinary guideline for diagnosis and treatment of basal cell carcinoma-update 2023. Eur J Cancer. 2023;192:113254. 37604067 10.1016/j.ejca.2023.113254

[12] Kazakov AS Zemskova MY Rystsov GK. Specific S100 proteins bind tumor necrosis factor and inhibit its activity. Int J Mol Sci . 2022;23:15956. 36555597 10.3390/ijms232415956 PMC9783754

[13] Lu C Liu J Yao M Li L Li G. Downregulation of S100 calcium binding protein A12 inhibits the growth of glioma cells. BMC Cancer. 2020;20:261. 32228516 10.1186/s12885-020-06768-7 PMC7106817

[14] Xia P Ji X Yan L Lian S Chen Z Luo Y. Roles of S100A8, S100A9 and S100A12 in infection, inflammation and immunity. Immunology. 2024;171:365–76. 38013255 10.1111/imm.13722

[15] Mints M Landin D Nasman A. Tumour inflammation signature and expression of S100A12 and HLA class I improve survival in HPV-negative hypopharyngeal cancer. Sci Rep. 2021;11:1782. 33469045 10.1038/s41598-020-80226-z PMC7815817

[16] Wang X Sun Z Tian W. S100A12 is a promising biomarker in papillary thyroid cancer. Sci Rep. 2020;10:1724. 32015423 10.1038/s41598-020-58534-1 PMC6997206

[17] Rassart E Desmarais F Najyb O Bergeron KF Mounier C. Apolipoprotein D. Gene. 2020;756:144874. 32554047 10.1016/j.gene.2020.144874 PMC8011330

[18] Sałkowska A Karaś K Karwaciak I. Identification of novel molecular markers of human Th17 cells. Cells. 2020;9:1611. 32635226 10.3390/cells9071611 PMC7407666

[19] Jankovic-Karasoulos T Bianco-Miotto T Butler MS. Elevated levels of tumour apolipoprotein D independently predict poor outcome in breast cancer patients. Histopathology. 2020;76:976–87. 31994214 10.1111/his.14081

[20] Sun L Zhao X Liu X. Transcription factor Ascl2 promotes germinal center B cell responses by directly regulating AID transcription. Cell Rep. 2021;35:109188. 34077723 10.1016/j.celrep.2021.109188

[21] Shang Y Jiang T Ran L. TET2-BCLAF1 transcription repression complex epigenetically regulates the expression of colorectal cancer gene Ascl2 via methylation of its promoter. J Biol Chem. 2022;298:102095. 35660018 10.1016/j.jbc.2022.102095 PMC9251787

[22] Orouji E Raman AT Singh AK. Chromatin state dynamics confers specific therapeutic strategies in enhancer subtypes of colorectal cancer. Gut. 2022;71:938–49. 34059508 10.1136/gutjnl-2020-322835 PMC8745382

[23] Yang Q Huang G Li L Li E Xu L. Potential mechanism of immune evasion associated with the master regulator ASCL2 in microsatellite stability in colorectal cancer. J Immunol Res. 2021;2021:5964752. 33628843 10.1155/2021/5964752 PMC7892217

[24] Xiao Y Yu D. Tumor microenvironment as a therapeutic target in cancer. Pharmacol Ther. 2021;221:107753. 33259885 10.1016/j.pharmthera.2020.107753 PMC8084948

[25] Liu C Chen B Huang Z Hu C Jiang L Zhao C. Comprehensive analysis of a 14 immune-related gene pair signature to predict the prognosis and immune features of gastric cancer. Int Immunopharmacol. 2020;89B(Pt):107074. 10.1016/j.intimp.2020.107074 33049494

[26] Chen T Yang C Dou R Xiong B. Identification of a novel 10 immune-related genes signature as a prognostic biomarker panel for gastric cancer. Cancer Med. 2021;10:6546–60. 34382341 10.1002/cam4.4180 PMC8446556

[27] Zhu MX Chen EB Yu S. Genomic profiling and the impact of MUC19 mutation in hepatoid adenocarcinoma of the stomach. Cancer Commun (Lond). 2022;42:1032–5. 35851588 10.1002/cac2.12336 PMC9558685

[28] Liu Z Wang A Pu Y. Genomic and transcriptomic profiling of hepatoid adenocarcinoma of the stomach. Oncogene. 2021;40:5705–17. 34326469 10.1038/s41388-021-01976-2

[29] Kang M Ma X Shi J. Distinct molecular phenotype and the potential prognostic value of immune prognostic index and tumor infiltrating lymphocytes in hepatoid adenocarcinoma of stomach. Transl Oncol. 2022;19:101380. 35276435 10.1016/j.tranon.2022.101380 PMC8908271

[30] Taniguchi Y Kiyozawa D Kohashi K. Volume of hepatoid component and intratumor M2 macrophages predict prognosis in patients with hepatoid adenocarcinoma of the stomach. Gastric Cancer. 2025;28:41–50 39488822 10.1007/s10120-024-01562-x PMC11706836

[31] Anderson NM Simon MC. The tumor microenvironment. Curr Biol. 2020;30:R921–5. 32810447 10.1016/j.cub.2020.06.081 PMC8194051

[32] Bejarano L Jordāo MJC Joyce JA. Therapeutic targeting of the tumor microenvironment. Cancer Discov. 2021;11:933–59. 33811125 10.1158/2159-8290.CD-20-1808

[33] Liu S Wang W Hu S. Radiotherapy remodels the tumor microenvironment for enhancing immunotherapeutic sensitivity. Cell Death Dis. 2023;14:679. 37833255 10.1038/s41419-023-06211-2 PMC10575861

[34] Taghizadeh-Hesary F. “Reinforcement” by tumor microenvironment: the seventh “R” of radiobiology. Int J Radiat Oncol Biol Phys. 2024;119:727–33. 38032584 10.1016/j.ijrobp.2023.09.027

