# Supplementary material for: Identification of a tumor microenvironment-related gene signature for predicting prognosis in patients with gastric cancer
Source: Medicine (Baltimore). 2025 Aug 29;104(35):e44032. doi: 10.1097/MD.0000000000044032 (PMC12401311; doi:10.1097/MD.0000000000044032)
Supplement: Supplementary file 1 [file medi-104-e44032-s001.docx]

| **Primer** | **Sequences** |
| --- | --- |
| CTHRC1 F | CTTACAAGGGCCAGCAGGAGT |
| CTHRC1 R | CTCAAAGCTTTCCCTCAGACATT |
| APOD F | tgagaactatgccctcgtgtatt |
| APOD R | actgtttctggagggagattagg |
| S100A12 F | caaaacttgaagagcatctggag |
| S100A12 R | caaaacttgaagagcatctggag |
| ASCL2 F | agctggtgaacttgggcttc |
| ASCL2 R | agctggtgaacttgggcttc |
| β-actin F | ctc cat cct ggc ctc gct gt |
| β-actin R | gct gtc acc ttc acc gtt cc |

**Table S1.** qRT-PCR primer sequence list.

| Antibody | Manufacturer | Cat No. | Host | Dilution |
| --- | --- | --- | --- | --- |
| CTHRC1 | proteintech | 16534-1-AP | Rabbit | 1:2000 |
| APOD | proteintech | 10520-1-AP | Rabbit | 1:2000 |
| S100A12 | proteintech | 16630-1-AP | Rabbit | 1:1000 |
| ASCL2 | proteintech | 21368-1-AP | Rabbit | 1:1000 |
| β-actin | HUABIO | HA722023 | Rabbit | 1:20,000 |

**Table S2. Antibodies used in Western blotting**
